# The Direction of Tumour Growth in Glioblastoma Patients

**DOI:** 10.1038/s41598-018-19420-z

**Published:** 2018-01-19

**Authors:** Morteza Esmaeili, Anne Line Stensjøen, Erik Magnus Berntsen, Ole Solheim, Ingerid Reinertsen

**Affiliations:** 1 Department of Circulation and Medical Imaging, NTNU, Norwegian University of Science and Technology, Trondheim, Norway; 2 Department of Radiology and Nuclear Imaging, St. Olavs University Hospital, Trondheim, Norway; 3 Department of Neurosurgery, St. Olavs University Hospital, Trondheim, Norway; 4 Norwegian National Advisory unit for Ultrasound and Image Guided Therapy, St. Olavs University Hospital, Trondheim, Norway; 5 Department of Neuromedicine and Movement Science, Faculty of Medicine, NTNU, Norwegian University of Science and Technology, Trondheim, Norway; 6 Department of Health Research, SINTEF Technology and Society, Trondheim, Norway

## Abstract

Generating MR-derived growth pattern models for glioblastoma multiforme (GBM) has been an attractive approach in neuro-oncology, suggesting a distinct pattern of lesion spread with a tendency in growing along the white matter (WM) fibre direction for the invasive component. However, the direction of growth is not much studied *in vivo*. In this study, we sought to study the dominant directions of tumour expansion/shrinkage pre-treatment. We examined fifty-six GBMs at two time-points: at radiological diagnosis and as part of the pre-operative planning, both with contrast-enhanced T1-weighted MRIs. The tumour volumes were semi-automatically segmented. A non-linear registration resulting in a deformation field characterizing the changes between the two time points was used together with the segmented tumours to determine the dominant directions of tumour change. To compute the degree of alignment between tumour growth vectors and WM fibres, an angle map was calculated. Our results demonstrate that tumours tend to grow predominantly along the WM, as evidenced by the dominant vector population with the maximum alignments. Our findings represent a step forward in investigating the hypothesis that tumour cells tend to migrate preferentially along the WM.

## Introduction

Derived from glial cells, glioblastoma multiforme (GBM) is an extremely aggressive malignant brain tumour affecting adults. Currently, the standard treatment offered to patients diagnosed with GBM combines surgery with chemo- and radio-therapy^1^. However, the treatment effectiveness is limited, as the disease continues to be almost universally fatal, with the median overall survival of only 12–18 months^2^.

After treatment, gliomas will usually relapse within 2-3 cm of the surgical resection cavity^3^. Already in 1938, Hans Joachim Scherer, a German neuropathologist, suggested from autopsy findings that gliomas migrate along existing brain structures triggered by the interaction of glioma cells with the neural microenvironment^4^. Glioma cells may migrate along blood vessels in the perivascular space or directly within the brain parenchyma^5^. Migration along blood vessels^6^ may be important for ensuring supply of oxygen and nutrition. However, despite promising effects in animal models, inhibition angioneogenesis with the anti–vascular endothelial growth factor (VEGF) antibody bevacizumab has not been able to improve survival in patients^7,8^. It has been suggested that glioma cell invasion may instead be triggered by hypoxia, perhaps explaining the disappointing results from clinical studies on angiogenesis inhibition so far^9^. Most studies on glioma cell migration are based on animal models, but glioma cell migration and invasion is often rather different from what is observed in humans. For example, a single cell migration is seldom observed in animal models^10^. At present, the mechanisms underlying glioblastoma growth and invasion are not fully understood, and human data are usually based on autopsy data and thus not longitudinal in nature.

As an abnormal capacity, the mobility of most of the cancer cells is greater than that of the normal cells^11^. Hence, it seems likely that direct movement is one of the mechanisms by which cancers spread through nearby tissues, resulting in random and heterogeneous growth patterns^12^. However, empirical evidence indicates that cancer cells can spread into some tissues more easily than into others. For example, large blood vessels characterized by very strong walls and dense tissues, such as cartilage, are harder for cancer cells to penetrate. This finding indicates that cancer cells grow along the path of least resistance, in line with most natural processes^13,14^. According to the generally accepted brain tumour development theory, tumour tends to grow along blood vessels or the white matter fibres, because of their anatomical characteristics, including a lubricated route for cancer cell migration^13,14^.

In the brain, water molecule diffuse along the white matter fibres in a directionally dependent manner. Magnetic resonance imaging (MRI) can map this diffusion phenomenon *in vivo* with the help of mathematical model and diffusion tensor imaging MR sequences. The diffusion tensor imaging (DTI) data demonstrate the direction of maximum diffusivity, providing a unique opportunity to study WM architecture^15^. Recently, MR-derived growth pattern models for GBM have been developed as an attractive approach in neuro-oncology, as they have revealed a distinct pattern of lesion spread with growth tendency along the white fibre direction for the invasive component. As such, a significant correlation was found between MR-derived tumour location and prognosis, and/or the region-specific genetic profile of tumour cells^16–18^.

Regardless of tumour grade, size or location, brain tumour patients frequently suffer from impairments in various cognitive domains, which are often difficult to explain based solely on the focal structural damage caused by a tumour. Some studies, therefore, highlight the importance of understanding glioma’s spreading patterns for pathophysiological impacts of brain tumours prior and after treatment^19–23^. The exact mechanisms behind tumour migration and growth are not completely understood, but from animal models and histopathological studies, it is seen that growth often follows the basal lamina of brain blood vessels or white matter tracts^24^. Thus, the direction of growth may be dependent on tumour location. Prediction of tumour growth directions may help define boundaries of focal treatments such as surgery or radiosurgery, thus improving the definition of the clinical target volume margins that are most critical for preventing future growth. For example, radiation target volumes should possibly be location specific, not just adding 2-3 cm in all directions as commonly done today. In addition, understanding and predicting growth, invasiveness and regrowth of glioblastoma in various locations of the brain may enhance prognostication, both in terms of predicting the location of tumour recurrences, but perhaps also for predicting future loss of functions as eloquent brain regions are invaded. So-called multifocal glioblastomas are presumably not truly multifocal but instead connected. Better models may be important for both targeting the most important portions of the tumour (e.g., the portion that leads to remote growth) and for avoiding both over- and under-treatment. A growth pattern model may further help classify tumours according to their degree of aggressiveness. In addition, the estimation of tumour growth pattern may be used for educational and scientific purposes.

In the current study, we aimed to investigate the prominent directions of GBM tumour growth inside the adult human brain. We also sought to validate the widely accepted theory postulating that brain tumour cells preferentially grow along WM fibre orientation by demonstrating that the disease targets intrinsic brain networks. We hypothesized that by using the MR images from 56 patients to compute the growth vector field derived from two pre-treatment time points, our understanding of generic solid tumour development and its interactions with local microenvironments may improve.

## Results

**Figure 1:**
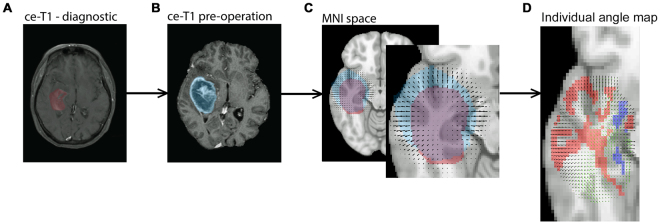
Schematic overview of the image analysis pipeline. (1) The diagnostic image (**A**) was registered to the pre-operative image (**B**), and the resulting transformation was applied to the tumour segmented from the diagnostic scan. (2) In the space of the pre-operative image, the tumours were masked out from images and registered non-linearly resulting in a local vector field characterizing the tumour growth pattern. (3) The pre-operative image was then registered to the MNI template to bring the pre-operative image with corresponding segmented tumour (blue mask) into standard MNI space. The resulting transforms were also applied to the diagnostic image with the corresponding segmented tumour (red mask) and the local vector field (**C**). Following this step all images, segmented tumours and local vector fields were in MNI space. (4) The individual vector fields were then compared to a DTI atlas to compute the voxel-wise alignment between tumour growth and white matter fibres (**D**). The vectors of deformation field (black colour) and the WM atlas (green colour). The predominant alignment between the deformation field vectors and the white matter fibres is visualized with color-coded voxels. Red voxels indicate that the tumour growth direction and the WM fibres are aligned (as determined by arbitrary thresholds - see the methods section), and the blue voxels indicate that the tumour growth direction and the WM fibres are perpendicular.

**Figure 2:**
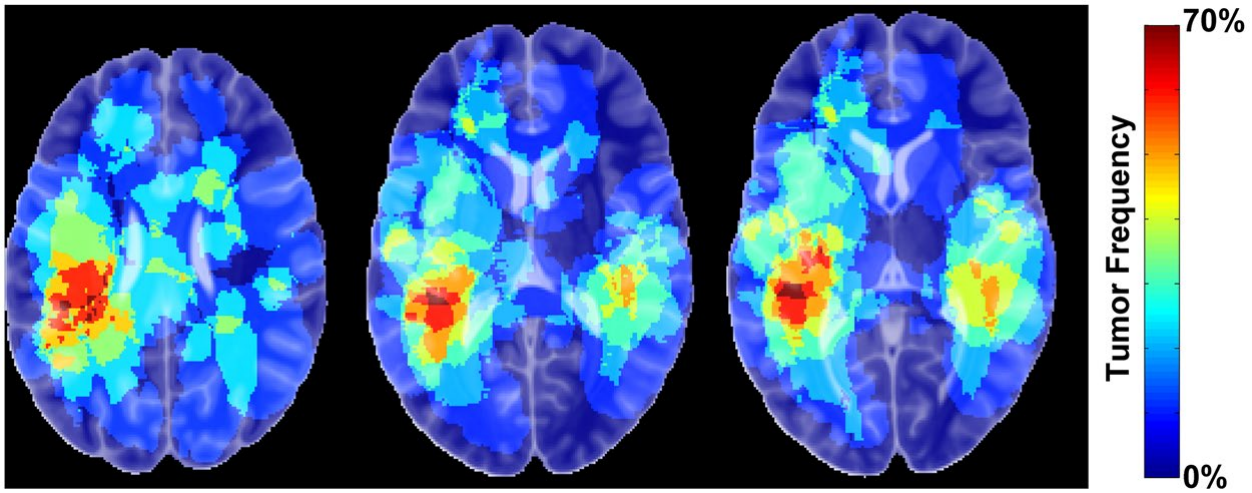
The frequency of tumour occurrence. Three axial slices are illustrated, indicating a predominance tumour occurrence within the temporal lobes.

**Figure 3:**
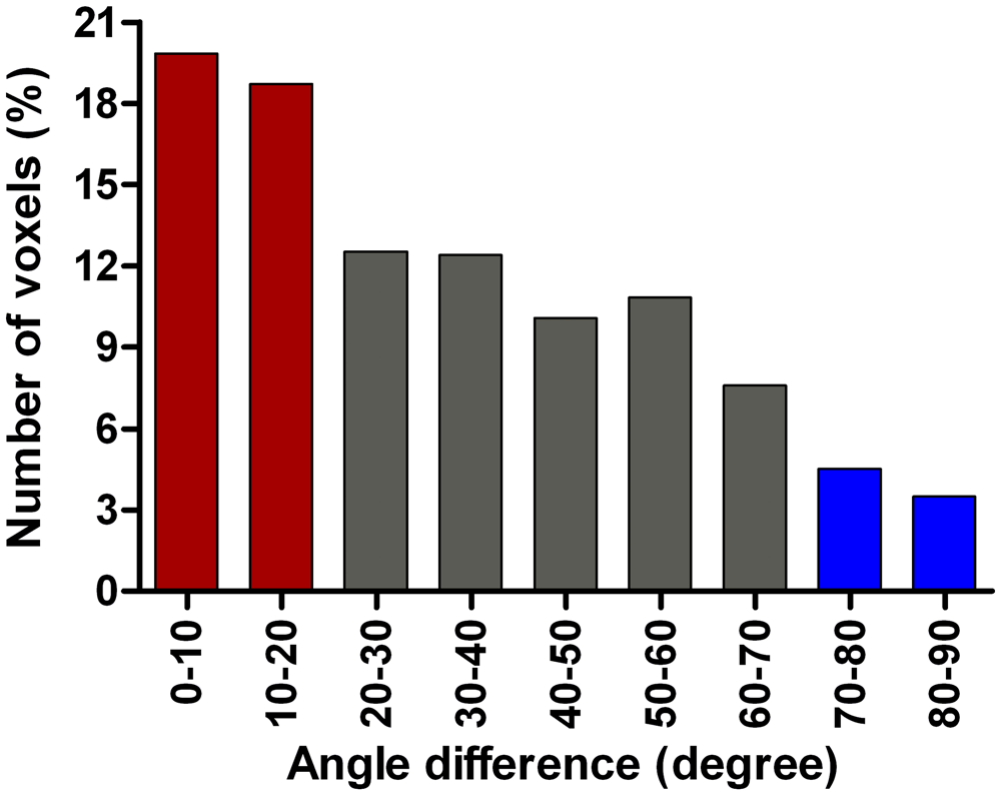
Distribution of angle differences between vectors of WM atlas and the deformation field. The distribution indicates that about 38.6% of total voxels with detectable vectors are aligned to that of corresponding voxels in the WM atlas (red bars), while about 8.02% were perpendicular (blue bars). The grey bars indicate the number of voxels that are out of the determined threshold ranges.

The image analysis pipeline described in the methods section (Fig. 1) were applied to the MR images from 56 patients that fulfilled the inclusion criteria. The tumour volumes measured in the pre-operative images were 2.5 ± 2.3 times (Mean ± SD) larger than the volumes measured in the diagnostic images. A predominance within the temporal lobes was observed in the tumour frequency analysis (Fig. 2). An axial slice of an individual angle map is shown in Fig. 1D. To depict the angle agreements, the scalar values of calculated angles were color-coded on the vector field, in which the maximum (parallel/anti-parallel) and the minimum (perpendicular) alignments were arbitrary determined as │θ│ < 20° and │θ│ > 70° respectively (Fig. 1D). Tumour measured in this study showed a tendency of moving along the white matter tracts, as evidenced by the dominant vector population with maximum alignments towards the tensor direction of the WM atlas (Fig. 3). This vector population reached 38.6%, by the determined thresholds (│θ│ < 20°), and 19.8% were clustered in the range of │θ│ < 10°. Of the total number of voxels, there were 8.02% of the voxels with vectors in the perpendicular direction (│θ│ > 70°), and 3.50% were clustered in the range of │θ│ > 80° (Fig. 3). The rest of the voxels were oriented in different directions outside the determined arbitrary thresholds. Growth parallel to the white matter fibers was significantly more common than the growth perpendicular to the fibers (Wilcoxon signed rank test, *p* = 0.014).

## Discussion

We demonstrate the growth directions of 56 GBM tumours with vector fields and show that the dominant growth direction of GBM is along the orientation of white matter fibres. The degree of alignment between the growth direction and white matter fibres was calculated on a voxel-wise basis using a DTI WM atlas. The resulting angle maps demonstrated that 38.6% of the vectors were aligned with the WM fibres. Although tumour growth in glioblastoma is more often seen along or parallel to the white matter tracts than perpendicular to them, a range of growth directions is observed. The percentage of parallel-to-anti-parallel vector population is independent of the variation of determined arbitrary thresholds, as the other angle ranges are equally distributed around both parallel and anti-parallel ranges (Fig. 3). Our findings represent a step forward in investigating the hypothesis that tumour cells tend to preferentially grow along the white matter fibres. The resulting vector fields of GBM tumour movements in various locations may provide useful information for future modeling of tumour growth prior to treatment.

Our result is in agreement with previous studies in the literature and provides additional support for the theory that cancer cells tend to grow along the path of least resistance. As a result of this growth pattern, GBMs interfere with global functional networks, rather than impacting the site of the lesion only. Therefore, several studies have been conducted in order to characterize network topology alterations in brain tumour patients before and after tumour resection^19^. To explore functional network topology of brain tumours, some authors used MR data acquired at resting state. Many studies have shown that disturbed network architecture of white matter, detected in pre-operative low-grade gliomas, may be associated with cognitive dysfunction^20,21^. Other approaches such as mathematical modelling were also employed to investigate tumour growth pattern^22,23^. The spread of glioma cells and adhesion mechanisms between glioma cells and the extracellular matrix (ECM) components were mathematically modelled using diffusion tensor imaging data^25,26^. The findings yielded by these studies indicate that the direction and drift velocity of tumour cells depend on the structure of white matter fibre network and the movement of glioma cells along the gradient of ECM density that surrounds the WM fibres.

This study has some limitations. We used a healthy DTI atlas for estimating the tumour orientations. This may decrease the accuracy of angle measures in some patients with larger tumour mass. A considerable tissue displacement may degrade the WM fibres morphology; therefore, the healthy DTI atlas may not precisely predict the WM orientations in the dataset. This source of error could be eliminated by using the patient-specific DTI. Since the data were acquired from different radiological centres, we unfortunately had no access to DTI images of these patients. However, this potential source of error was reduced by including relatively large patient cohorts in this study. In addition, using a publicly accessible DTI atlas makes the investigation pipeline reproducible for feature studies.

In conclusion, the results of the current study suggest that the dominant growth direction of GBM tumour is along of the white matter fibres, as visualized with an angle map and a comparison between the population of parallel and perpendicular vectors. The prediction of tumour growth direction in various locations of the brain may greatly assist in the modeling of tumour growth prior to treatment. A similar study on lower-grade gliomas and a larger cohort can be performed to investigate the feasibility of an accurate modeling of tumour growth direction.

## Methods

### Population Data

Patients diagnosed with glioblastoma between January 2004 and May 2014 were retrospectively evaluated for inclusion, based on the following eligibility criteria: age ≥18 years, no prior history of brain tumour and having 2 contrast-enhanced T1-weighted (ce-T1) MRI examinations with the time interval >14 days (Mean ± standard deviation (SD) = 25 ± 9 days) to minimize the uncertainty in the growth rate estimates^27^, and comparable image quality and spatial resolution acquired at both time points to ensure the accuracy of deformation field estimation. A total number of 56 patients met the inclusion criteria. The patients were examined at initial diagnosis, and prior to surgery for intraoperative neuronavigation. The study was approved by the Regional Ethics Committee of Central Norway and adhered to the Helsinki Declaration. All patients provided written informed consent to participate.

### Magnetic Resonance Imaging Protocols

MRI data were acquired either at a 1 T, 1.5 T or 3 T MR systems. The system’s standard 32-channel phased-array head coil was used for imaging. The diagnostic scans were performed in one of 12 different radiology clinics, located in the geographical region of our neurosurgical department. All preoperative scans were performed at our hospital. Gadolinium-based contrast agents were used for ce-T1 imaging. For the diagnostic scans, the following protocol parameters were used: repetition time (TR) = 4500ms, echo time (TE) = 14ms, slice thickness of 0.6–1.0 mm (54 patients) and 5 mm (12 patients), in-plane resolution from 0.45 × 0.45 to 1.0 × 1.0 mm^2^, 19 slices, and 224 × 246 to 512 × 512 matrix dimension. Pre-operation ce-T1 images acquired with TR/TE = 10000/70 ms, slice thickness of either 1.0 mm (7 patients) or 1.5 mm (49 patients), in-plane resolution of 0.5 × 0.5 mm^2^, 23 slices, and 512 × 512 matrix size.

### Tumour Segmentation

Contrast-enhanced T1-weighted images from both diagnostic and pre-operative examinations were used for tumour delineation and segmentation. The image analysis software BrainVoyager QX (Version 2.3, Brain Innovation B.V., Maastricht, The Netherlands) was used for semi-automatic tumour segmentation. One of the observers, who was blinded to the subjects’ identity, performed delineations of Regions of interests (ROIs). Inside these ROIs, the tumors were segmented using a combination of intensity thresholding and manual delineation. The final segmentations were verified by a neuroradiologist (E. M. B.). Tumor volumes were calculated from the voxel volume and the number of voxels segmented. In some cases, the diagnostic images had gaps between slices, and the voxel volume was calculated as voxel-in-plane-resolution × (slice thickness + gap thickness) as previously described in detail^27^. Tumour volume was defined as the contrast-enhancing compartment of the tumour combined with the central non-enhancing compartment enclosed by the contrast (the latter usually represented necrosis).

### Pipeline for Image Registration and Deformation Field Estimation

To obtain tumour deformation fields, we used widely adopted registration methods to estimate the tumour changes between the diagnosis and pre-operation time points. Advanced Normalization Tools (ANTs) script packages^28^ and MATLAB (MathWorks, Natick, MA, USA) were used for registration of MR images. The data analysis pipeline (Fig. 1) commences with resampling of the MR images to obtain identical MR voxel size (all images were resampled to 1 × 1 × 1 mm^3^) in both diagnostic and pre-operative MR images. The N4 bias field correction, available in the ANTs package, was applied to all MR data to minimize the field inhomogeneity effects and artificial low-frequency intensity variations across the images^28^. The N4 bias correction is an improved version of the non-parametric non-uniform intensity normalization (N3) algorithm with an enhanced B-spline least squares fitting routine (which includes multi-resolution capabilities) and a modified optimization formulation for improved bias field correction. MR images were skull-stripped using the Brain Extraction Tool (BET) algorithm^29^ provided in FSL package (https://fsl.fmrib.ox.ac.uk/fsl/). To estimate the deformation of tumour volumes, we performed both linear and non-linear registrations using ANTs tools.

First, the affine registration from the ANTs package was used to align the diagnostic with the pre-operative image for each patient. The resulting affine transformation matrix was then applied to the segmented tumour from the diagnostic image. Both MR images (diagnostic and pre-operative) with corresponding tumour segmentations (binary masks) were thus aligned in the space of pre-operative MR image. Second, the tumour regions were masked out from the diagnostic and pre-operative images respectively. Using the non-linear registration tool provided by ANTs, which is based on a b-spline function, the tumour region from the diagnostic image was registered to the tumour region from the pre-operative image. This process yielded a localized deformation field, characterizing the changes in tumour shape and volume between the two time points.

The resulting deformation fields were projected into a common space using the MNI152 template. The MNI152 template is an average of structural MR images acquired from 152 healthy individuals and registered to a common space^30^. The registration to MNI space was performed using affine registration in ANTs, between the pre-operative image and the MNI template. To investigate the degree of alignment between the individual vector fields and the white matter fibres, a DTI white matter (WM) atlas^31^ was used. The atlas is constructed from the IXI brain database (http://www.ixi.org.uk) and consists of both tensor intensities and vectors information (https://www.nitrc.org/). An affine transformation was computed to align the DTI volume to MNI space as well. Next, the deformation field derived from all patients were down-sampled to the voxel size of the DTI atlas (1.75 × 1.75 × 2.25 mm^3^). This allowed us to calculate the angles between the corresponding vectors obtained from each voxel in the deformation field space and the DTI atlas per patient. The angle maps were generated in 3D space, representing voxel-wise θ values using the dot product (equation 1).

1 $${\boldsymbol{\theta }}={\boldsymbol{co}}{{\boldsymbol{s}}}^{-1}(\frac{{\boldsymbol{u}}\cdot {\boldsymbol{v}}}{\parallel {\boldsymbol{u}}\parallel \parallel {\boldsymbol{v}}\parallel })\,$$

where the angle between vectors u and v is calculated by the inverse cosine of the dot product of the vectors, divided by the product of each of their magnitudes. To simplify the angle map visualization, we remapped the scalar angle value to the range from 0 ° to 90 °, ignoring the matrix sign of the vectors.

We also calculated the spatial frequency of tumour occurrence in our cohort, by adding the tumour volumes extracted from the pre-operative images and co-registered into MNI space. The generated tumour frequency was color-coded to demonstrate the degree of tumor spatial overlap among the patients.

### Statistics

Statistical analyses were performed using GraphPad Prism (GraphPad Software, Inc. V 4.03, CA, USA). Voxel populations in the angle map were compared across the determined thresholds; parallel (│θ│ < 20°) versus perpendicular (│θ│ > 70°), using the non-parametric Wilcoxon signed rank test with the threshold for statistical significance defined as *p* *≤* 0.05.
